# Linkage disequilibrium of single-nucleotide polymorphism data: how sampling methods affect estimates of linkage disequilibrium

**DOI:** 10.1186/1753-6561-3-s7-s105

**Published:** 2009-12-15

**Authors:** Qimei He, Bradley J Willcox

**Affiliations:** 1 Pacific Health Research Institute, 700 Bishop Street, Bishop Tower, Suite 900, Honolulu, Hawaii 96813 USA; 2 Queen's Medical Center, Honolulu, Hawaii 96813 USA; 3 Department of Geriatric Medicine, John A. Burns School of Medicine, University of Hawaii, Honolulu, Hawaii 96817 USA

## Abstract

Linkage disequilibrium (LD) is an important measure used in the analysis of single-nucleotide polymorphism (SNP) data. We used the Genetic Analysis Workshop 16 (GAW16) Framingham Heart Study 500 k SNP data to explore the effect of sampling methods on estimating of LD for SNP data.

**Method and data:**

We found 332 trios in the GAW16 Framingham SNP data. Repeated random samples without replacement, of different sizes of trios and independent individuals, are drawn from these 332 trios. For each sample, the LD is calculated using the Haploview program for the chromosome 1 SNP data. Percents of *D*' > 0.8 and *r*^2 ^> 0.8 are calculated for different distance bins based on the Haploview output. The results are summarized by sample size and sampling methods to give us an overall view of the effect of sample size and sampling methods on the LD estimation.

**Results:**

Trios design gave stable estimates. A sample of 30 to 40 trios gave estimates of percent of LD > 0.8 very close to those from 332 trios. When independent individuals are used, the estimates are less stable and are different from those obtained from the 332 trios for both *D*' and *r*^2^, with larger differences for *D*'.

**Conclusion:**

Our results suggest that trio design gives a stable estimate of LD. Therefore it may be more suitable for LD analysis than using independent individuals. We must be cautious when comparing the LD estimates from trios, and those from independent individuals.

## Background

Linkage disequilibrium (LD) is an important measure in analyzing single-nucleotide polymorphism (SNP) data. It helps us to understand the evolutionary history of humans and other organism. A thorough understanding of LD also helps us to better design and analyze studies of SNP-disease associations.

Two major sampling methods are used in the estimate of LD. One method uses trios in the analysis. Each trio has a pair of parents and one child. The other uses independent individuals. Both methods use an expectation-maximization algorithm to estimate the haplotype, and use the estimated haplotype in the LD calculation. However, the trio design uses the parents' SNP genotype in the analysis to infer the haplotype of the child. It is possible that this additional information makes the estimated haplotype more accurate. Fallin and Schork [1] examined the accuracy of haplotype estimate using unphased diploid data collected from individuals and concluded that the estimated haplotype frequencies are quite accurate. When summarizing their findings, they pointed out that "any statistical-inference procedures that make use of haplotype frequency estimates demands independent attention."

In November 2004, the HapMap Project [2] released whole-genome SNP genotype data for four major populations: 30 trios for Caucasian, 30 trios for African, 45 independent individuals for Chinese, and 45 independent individuals for Japanese. These data are widely used in the genetic research communities to assess the LD for these four populations and for other purposes. For example, Bonnen et al. [3] used these four HapMap populations and a sample of 30 trios from Kosrae to compare the LD patterns of these populations and to assess the feasibility of whole-genome association study using the Kosrae population. This year, HapMap Project released its HapMap 3 SNP data, which includes 90 to 180 persons in each of 11 populations. Some population had both trios and independent individuals, some have only trios, and Chinese and Japanese populations had only independent individuals.

In the near future, HapMap 3 will be one of the major SNP data sources for the genetic research communities to assess the LD pattern in different populations. In this case we have to ask questions concerning the use of different designs (trios or independent individual) and the sample sizes in LD estimation. For example, do the trios and independent individuals offer compatible estimate of LD? How does sample affect the estimate of LD? There are no answers to these questions in the literature. Therefore, we used the Genetic Analysis Workshop 16 (GAW16) Framingham Heart Study 500 k SNP data to examine the effects of the sampling methods and sample size on the estimate of LD for SNP data.

## Methods

### GAW16 Framingham Heart Study data

The GAW16 Framingham Heart Study 500 k SNP data set has genotype data for more than 6500 individuals from three cohorts of the Framingham Heart Study. Based on the family structure offered by the workshop, we identified 332 trios with SNP genotype data. All of these 332 trios are either from different families, or from the same family but share no common ancestors. Therefore, we can assume that they are independent.

### Analysis

Based on these 332 trios, 80 random samples of trios were drawn without replacement for sample sizes of 25, 30, 40, 50, 100, 200, and 300. We also used offspring in these trios to form a pool of 332 independent individuals. Eighty random samples of independent individuals for sample sizes of 25, 30, 40, 50, 100, 200, and 300 were drawn without replacement from this pool. The Haploview program [4] was used to calculate the LD measures *D*' and *r*^2 ^for each random sample for all SNP pairs within a distance of 1000 kb on chromosome 1. We also calculated *D*' and *r*^2 ^for SNP pairs for the 332 trios in the same way. Then percent of *D*'> 0.8 and *r*^2 ^> 0.8 were calculated for different distance bins for each sample. The mean, standard deviation, and range of percent of LD measures greater than 0.8 among the repeated random samples were calculated for each sample size for trios and independent individuals. These results were compared with those obtained from the 332 trios. This gives us a picture how the sample size and sampling design (trio or independent individuals) affect the estimate of the LD.

## Results

The results are presented in four tables. Table 1 presents the percent of *D*' > 0.8 for different distance bins for the trio design. Table 2 presents the percent of *D*' > 0.8 for different distance bins for independent individuals design. Tables 3 and 4 present the percent of *r*^2 ^> 0.8 for different distance bins for trios design and independent individual design, respectively.

**Table 1:** Percent of *r*^2 ^> 0.8 of 80 random samples of trios by sample size, compared to percent of *r*^2 ^> 0.8 for the 332 trios

Distance bin (kb)	Range and mean ± SD								
		
	*n *= 25	*n *= 30	*n *= 40	*n *= 50	*n *= 100	*n *= 150	*n *= 200	*n *= 300	332 trios
0 - 5	26.0-27.4	27.1-28.4	27.0-28.3	27.2-28.3	27.2-28.3	27.3-28.1	27.3-28.0	27.3-27.9	27.7
	26.8 ± 0.3	27.9 ± 0.3	27.8 ± 0.2	27.8 ± 0.3	27.7 ± 0.2	27.7 ± 0.2	27.7 ± 0.2	27.7 ± 0.1	
5 - 10	15.8-16.9	16.7-17.7	16.5-17.5	16.6-17.4	16.6-17.4	16.6-17.4	16.6-17.3	16.6-17.2	17.1
	16.5 ± 0.2	17.1 ± 0.2	17.0 ± 0.2	17.0 ± 0.2	17.0 ± 0.2	17.0 ± 0.2	17.0 ± .15	17.0 ± 0.1	
10 - 15	11.5-12.5	12.0-12.9	12.1-12.8	12.0-12.8	12.0-12.8	12.0-12.7	12.0-12.6	12.1-12.6	12.4
	12.1 ± 0.2	12.5 ± 0.2	12.4 ± .15	12.4 ± 0.2	12.4 ± 0.2	12.4 ± 0.1	12.4 ± .11	12.4 ± 0.1	
15 - 20	8.94-9.82	9.41-10.3	9.38-10.2	9.30-10.1	9.37-10.2	9.34-9.94	9.32-10.0	9.59-9.88	9.84
	9.48 ± 0.17	9.81 ± .17	9.72 ± .15	9.72 ± .17	9.69 ± 0.17	9.71 ± 0.13	9.73 ± .12	9.75 ± .07	
20 - 25	6.94-7.67	7.17-7.92	7.13-7.75	7.17-7.87	7.25-7.77	7.27-7.79	7.26-7.75	7.38-7.70	7.61
	7.33 ± 0.15	7.57 ± .16	7.50 ± .13	7.51 ± .14	7.51 ± 0.12	7.51 ± 0.10	7.49 ± .11	7.55 ± 0.07	
25 - 40	4.77-5.31	4.83-5.40	4.84-5.29	4.89-5.35	4.84-5.35	4.87-5.34	4.85-5.32	4.90-5.14	5.07
	5.04 ± 0.11	5.16 ± .12	5.08 ± .08	5.10 ± .10	5.07 ± 0.11	5.08 ± 0.08	5.06 ± 0.08	5.04 ± 0.5	
40 - 60	2.78-3.13	2.84-3.16	2.78-3.14	2.83-3.13	2.83-3.12	2.84-3.14	2.78-3.07	2.85-2.96	2.88
	2.96 ± 0.08	3.00 ± .08	2.97 ± .07	2.98 ± .07	2.97 ± 0.07	2.97 ± 0.06	2.95 ± 0.05	2.90 ± .02	
60 - 80	1.64-1.89	1.64-1.95	1.63-1.87	1.62-1.90	1.59-1.85	1.64-1.87	1.63-1.84	1.68-1.77	1.73
	1.78 ± .06	1.78 ± 0.6	1.75 ± .05	1.75 ± .05	1.74 ± .05	1.76 ± .04	1.75 ± 0.04	1.73 ± 0.02	
80 - 100	1.10-1.36	1.11-1.32	1.11-1.29	1.09-1.28	1.08-1.26	1.09-1.26	1.10-1.27	1.12-1.19	1.15
	1.23 ± 0.05	1.22 ± .04	1.19 ± .04	1.20 ± 0.4	1.18 ± 0.04	1.18 ± 0.03	1.18 ± .03	1.15 ± .02	
100 - 120	0.72-0.89	0.70-0.88	0.68-0.86	0.70-0.85	0.66-0.82	0.68-0.82	0.67-0.82	0.72-0.78	0.74
	0.81 ± 0.04	0.78 ± 0.3	0.77 ± .03	0.77 ± .03	0.76 ± 0.03	0.75 ± .03	0.75 ± .03	0.75 ± 0.01	
120 - 140	0.51-0.66	0.50-0.63	0.48-0.61	0.49-0.62	0.47-0.62	0.50-0.61	0.49-0.61	0.52-0.58	0.54
	0.59 ± 0.03	0.57 ± 0.3	0.55 ± .03	0.56 ± .03	0.55 ± 0.03	0.55 ± 0.02	0.55 ± .02	0.55 ± 0.01	
140 - 160	0.33-0.45	0.32-0.44	0.32-0.41	0.31-0.43	0.32-0.43	0.32-0.40	0.30-0.40	0.33-0.37	0.34
	0.39 ± 0.02	0.37 ± .02	0.36 ± .02	0.36 ± .02	0.36 ± .02	0.35 ± 0.02	0.35 ± 0.02	0.35 ± 0.01	
160 - 180	0.25-0.35	0.22-0.33	0.24-0.32	0.24-0.32	0.24-0.32	0.23-0.30	0.24-0.32	0.25-0.28	0.26
	0.30 ± 0.02	0.28 ± .02	0.28 ± .02	0.27 ± .02	0.28 ± .02	0.27 ± 0.02	0.27 ± .01	0.27 ± 0.01	
180 - 200	0.17-0.25	0.16-0.24	0.16-0.23	0.16-0.23	0.16-0.23	0.17-0.22	0.16-0.24	0.18-0.21	0.19
	0.22 ± 0.02	0.20 ± .02	0.19 ± .01	0.20 ± .01	0.20 ± 0.01	0.19 ± 0.01	0.19 ± 0.01	0.19 ± 0.01	

**Table 2:** Percent of *r*^2 ^> 0.8 of the 80 random samples of independent individuals by sample size, compared to percent of *r*^2 ^> 0.8 for the 332 trios

Distance bin (kb)	Range and mean ± SD								
		
	*n *= 25	*n *= 30	*n *= 40	*n *= 50	*n *= 100	*n *= 150	*n *= 200	*n *= 300	332 trios
0 - 5	26.9-28.5	26.7-28.1	26.0-27.4	23.7-26.8	25.4-27.0	26.6-27.0	26.7-27.1	26.9-27.1	27.7
	27.7 ± 0.3	27.4 ± 0.3	26.7 ± 0.2	26.1 ± 0.7	26.4 ± 0.4	26.8 ± 0.3	26.9 ± 0.1	27.0 ± 0.04	
5 - 10	16.8-17.9	16.5-17.5	15.8-16.8	14.0-16.6	15.3-16.5	16.0-16.6	16.1-16.4	16.3-16.5	17.1
	17.2 ± 0.2	16.9 ± 0.2	16.4 ± 0.2	15.9 ± 0.5	16.0 ± 0.3	16.3 ± 0.2	16.3 ± 0.1	16.4 ± 0.04	
10 - 15	12.4-13.3	12.1-13.0	11.6-12.5	10.1-12.2	11.0-12.0	11.5-12.0	11.6-11.9	11.8-11.9	12.4
	12.8 ± 0.2	12.5 ± 0.2	12.0 ± 0.2	11.6 ± 0.4	11.6 ± 0.2	11.7 ± 0.2	11.8 ± 0.1	11.8 ± 0.04	
15 - 20	9.72-10.7	9.52-10.3	9.06-9.79	7.94-9.53	8.45-9.33	8.91-9.34	9.02-9.28	9.09-9.27	9.84
	10.1 ± 0.2	9.86 ± 0.18	9.41 ± 0.14	9.06 ± 0.38	9.00 ± 0.20	9.14 ± 0.20	9.15 ± 0.06	9.18 ± 0.04	
20 - 25	7.68-8.40	7.34-8.10	7.06-7.65	6.06-7.47	6.46-7.19	6.89-7.22	6.94-7.16	7.02-7.15	7.61
	7.95 ± 0.19	7.73 ± 0.15	7.36 ± 0.12	6.98 ± 0.30	6.86 ± 0.18	7.04 ± 0.17	7.05 ± 0.05	7.08 ± 0.03	
25 - 40	5.24-5.70	5.07-5.52	4.85-5.21	4.14-5.10	4.22-4.90	4.64-4.83	4.68-4.82	4.72-4.82	5.07
	5.50 ± 0.10	5.33 ± 0.09	5.03 ± 0.08	4.75 ± 0.20	4.62 ± 0.13	4.75 ± 0.11	4.75 ± 0.03	4.78 ± 0.02	
40 - 60	3.09-3.52	3.00-3.41	2.84-3.17	2.44-3.11	2.44-2.86	2.70-2.85	2.70-2.81	2.73-2.79	2.88
	3.31 ± 0.08	3.19 ± 0.09	2.99 ± 0.07	2.81 ± 0.13	2.70 ± 0.09	2.77 ± 0.07	2.76 ± 0.02	2.76 ± 0.01	
60 - 80	1.91-2.25	1.84-2.13	1.72-1.96	1.48-1.89	1.41-1.68	1.58-1.69	1.55-1.66	1.59-1.65	1.73
	2.06+0.07	1.97 ± 0.07	1.83 ± 0.06	1.69 ± 0.08	1.57 ± 0.06	1.63 ± 0.05	1.61 ± 0.2	1.62 ± 0.01	
80 - 100	1.30-1.58	1.25-1.50	1.18-1.34	1.05-1.28	0.97-1.17	1.06-1.17	1.07-1.15	1.09-1.13	1.15
	1.43 ± 0.05	1.36. ± 05	1.26 ± 0.04	1.17 ± 0.05	1.08 ± 0.04	1.11 ± 0.03	1.11 ± 0.02	1.11 ± 0.01	
100 - 120	0.87-1.10	0.83-1.02	0.77-0.94	0.69-0.92	0.60-0.79	0.66-0.74	0.67-0.73	0.68-0.72	0.74
	0.98 ± 0.05	0.92 ± 0.04	0.84 ± 0.04	0.79 ± 0.04	0.71 ± 0.04	0.71 ± 0.03	0.70 ± 0.01	0.70 ± 0.009	
120 - 140	0.62-0.83	0.60-0.75	0.55-0.69	0.47-0.63	0.39-0.57	0.46-0.54	0.46-0.53	0.48-0.52	0.54
	0.72 ± 0.04	0.68 ± 0.03	0.62 ± 0.03	0.57 ± 0.03	0.50 ± 0.03	0.51 ± 0.02	0.50 ± 0.01	0.49 ± 0.007	
140 - 160	0.42-0.57	0.41-0.53	0.37-0.47	0.32-0.48	0.28-0.37	0.30-0.37	0.32-0.36	0.32-0.36	0.34
	0.49 ± 0.03	0.46 ± 0.03	0.42 ± 0.02	0.38 ± 0.03	0.32 ± 0.02	0.34 ± 0.02	0.34 ± 0.01	0.34 ± 0.006	
160 - 180	0.31-0.44	0.30-0.41	0.26-0.39	0.24-0.39	0.20-0.27	0.23-0.28	0.23-0.28	0.24-0.26	0.26
	0.39 ± 0.03	0.36 ± 0.02	0.32 ± 0.02	0.29 ± 0.03	0.24 ± 0.02	0.25 ± 0.01	0.25 ± 0.01	0.25 ± 0.004	
180 - 200	0.23-0.36	0.20-0.33	0.18-0.31	0.18-0.34	0.13-0.21	0.16-0.20	0.15-0.20	0.16-0.18	0.19
	0.29 ± 0.03	0.27 ± 0.02	0.24 ± 0.02	0.22 ± 0.04	0.17 ± 0.01	0.18 ± 0.1	0.17 ± 0.01	0.17 ± 0.004	

**Table 3:** Percent of *D*' > 0.8 of 80 random samples of trios by sample size, compared to percent of *D*' > 0.8 for the 332 trios

Distance bin (kb)	Range and mean ± SD								
		
	*n *= 25	*n *= 30	*n *= 40	*n *= 50	*n *= 100	*n *= 150	*n *= 200	*n *= 300	332 trios
0 - 5	90.5-91.9	89.7-91.1	90.0-91.1	89.9-91.1	89.8-91.2	90.0-91.2	90.1-91.1	90.5-91.1	90.7
	91.3 ± 0.3	90.6 ± 0.3	90.5 ± 0.2	90.5 ± 0.3	90.6 ± 0.3	90.6 ± 0.2	90.7 ± 0.2	90.8 ± 0.1	
5 - 10	80.6-82.0	78.9-80.4	79.1-80.3	79.1-80.6	79.0-80.2	79.1-80.3	79.3-80.2	79.3-80.1	79.6
	81.5 ± 0.3	79.8 ± 0.3	79.6 ± 0.3	79.7 ± 0.3	79.7 ± 0.3	79.6 ± 0.2	79.7 ± 0.2	79.6 ± 0.2	
10 - 15	74.3-75.6	71.6-73.3	71.7-73.2	71.5-73.1	71.6-73.0	71.5-73.1	71.6-72.6	71.4-72.3	71.9
	74.9 ± 0.3	72.5 ± 0.4	72.3 ± 0.3	72.8 ± 0.3	72.3 ± 0.3	72.1 ± 0.3	72.1 ± 0.2	71.9 ± 0.1	
15 - 20	68.9-70.5	65.7-67.6	65.7-67.1	65.4-67.3	65.6-67.1	65.6-66.8	65.8-66.7	65.9-66.5	66.2
	68.9 ± 0.3	66.6 ± 0.4	66.3 ± 0.3	66.3 ± 0.3	66.3 ± 0.3	66.2 ± 0.3	66.3 ± 0.2	66.2 ± 0.1	
20 - 25	64.0-65.5	60.3-62.1	60.2-61.4	60.1-61.5	59.8-61.5	60.0-61.5	59.6-61.4	60.7-61.4	61.1
	64.6 ± 0.3	61.1 ± 0.3	60.8 ± 0.3	60.9 ± 0.3	60.7 ± 0.3	60.7 ± 0.3	60.6 ± 0.3	61.0 ± 0.1	
25 - 30	59.7-61.4	56.0-57.4	55.6-57.0	55.6-57.2	55.6-57.0	55.7-57.3	55.5-56.8	56.1-56.9	56.6
	60.4 ± 0.3	56.7 ± 0.4	56.3 ± 0.3	56.4 ± 0.4	56.3 ± 0.3	56.3 ± 0.3	56.3 ± ± 0.3	56.5 ± 0.1	
30 - 40	54.5-56.0	50.1-51.5	49.7-51.1	49.8-51.4	49.6-50.9	49.7-51.1	49.9-51.2	50.2-51.0	50.6
	55.3 ± 0.3	50.8 ± 0.3	50.4 ± 0.3	50.5 ± 0.3	50.4 ± 0.3	50.5 ± 0.2	50.5 ± 0.3	50.5 ± 0.2	
40 - 50	49.3-50.9	44.1-45.6	43.4-45.4	43.9-45.2	43.8-45.2	43.8-45.2	43.6-45.1	44.1-45.0	44.5
	49.9 ± 0.3	44.9 ± 0.3	44.4 ± 0.3	44.5 ± 0.3	44.4 ± 0.3	44.5 ± 0.3	44.3 ± 0.3	44.5 ± 0.2	
50 - 60	45.5-47.3	39.9-41.4	39.5-41.0	39.5-41.2	39.5-40.9	39.6-41.0	39.6-40.7	39.8-40.5	40.2
	46.4 ± 0.4	40.6 ± 0.3	40.1 ± 0.3	40.2 ± 0.3	40.1 ± 0.3	40.2 ± 0.3	40.1 ± 0.3	40.2 ± 0.1	
60 - 70	42.4-44.1	36.5-37.8	36.1-37.4	35.9-37.3	35.7-37.3	35.9-37.5	36.0-37.2	362-37.1	36.9
	43.2 ± 0.3	37.2 ± 0.3	36.60.3	36.6 ± 0.3	36.6 ± 0.3	36.6 ± 0.3	36.6 ± 0.3	36.7 ± 0.2	
70 - 80	39.7-41.3	33.1-34.6	32.9-34.0	32.7-34.1	32.7-34.0	32.7-34.1	32.9-34.1	33.1-33.9	33.6
	40.3 ± 0.4	33.9 ± 0.3	33.4 ± 0.3	33.4 ± 0.3	33.4 ± 0.3	33.5 ± 0.3	33.5 ± 0.2	33.6 ± 0.1	
80 - 90	37.5-39.5	31.1-32.9	30.7-32.0	30.5-32.4	30.6-31.7	30.7-31.8	30.7-31.8	30.8-31.7	31.2
	38.4 ± 0.4	31.8 ± 0.4	31.2 ± 0.3	31.3 ± 0.4	31.1 ± 0.3	31.2 ± 0.3	31.2 ± 0.2	31.1 ± 0.2	
90 - 100	35.4-37.4	28.7-30.4	28.2-29.7	28.2-30.1	28.0-29.5	28.2-29.7	28.3-29.4	28.5-29.2	28.8
	36.4 ± 0.4	29.5 ± 0.4	28.9 ± 0.3	29.0 ± 0.4	28.9 ± 0.3	28.8 ± 0.3	28.8 ± 0.2	28.8 ± 0.1	
100 - 140	32.3-34.1	25.3-26.4	24.6-25.8	24.7-25.9	24.7-25.7	24.6-25.8	24.9-25.9	25.3-25.9	25.7
	33.1 ± 0.4	25.9 ± 0.3	25.3 ± 0.2	25.3 ± 0.3	25.2 ± 0.2	25.3 ± 0.2	25.4 ± 0.2	25.6 ± 0.1	
140 - 180	28.8-30.5	21.5-22.7	21.0-21.8	20.8-22.0	20.9-21.7	20.8-22.0	20.8-21.8	21.1-21.7	21.4
	29.7 ± 0.4	22.1 ± 0.2	21.4 ± 0.2	21.4 ± 0.3	21.3 ± 0.2	21.4 ± 0.2	21.4 ± 0.2	21.4 ± 0.1	

**Table 4:** Percent of *D*' > 0.8 of 80 random samples of independent individuals by sample size, compared to percent of *D*' > 0.8 for the 332 trios

Distance bin (kb)	Range and mean ± SD								
		
	*n *= 25	*n *= 30	*n *= 40	*n *= 50	*n *= 100	*n *= 150	*n *= 200	*n *= 300	332 trios
0 - 5	88.4-89.5	88.1-89.2	87.5-88.6	86.6-88.3	85.0-86.9	85.8-86.8	85.9-86.6	86.1-86.8	90.7
	89.0 ± 0.3	88.6 ± 0.3	88.1 ± 0.3	87.8 ± 0.4	86.1 ± 0.4	86.2 ± 0.4	86.3 ± 0.1	86.6 ± 0.1	
5 - 10	80.3-81.9	79.2-81.1	78.5-80.0	77.7-79.6	74.5-76.6	74.6-75.8	74.6-75.3	74.7-75.3	79.6
	81.0 ± 0.3	80.3 ± 0.3	79.3 ± 0.3	78.7 ± 0.4	75.7 ± 0.5	75.2 ± 0.5	75.0 ± 0.2	75.0 ± 0.1	
10 - 15	75.3-76.7	74.3-75.8	72.9-74.4	71.9-73.7	67.9-69.9	67.4-68.5	67.1-67.9	67.1-76.7	71.9
	76.0 ± 0.3	75.1 ± 0.3	73.7 ± 0.3	73.0 ± 0.4	69.0 ± 0.5	67.9 ± 0.6	67.6 ± 0.2	67.3 ± 0.1	
15 - 20	71.2-72.8	70.1-71.5	68.5-69.8	67.1-69.1	61.8-64.1	61.5-62.4	61.0-61.9	60.7-61.2	66.2
	72.0 ± 0.3	70.8 ± 0.3	69.2 ± 0.3	68.1 ± 0.4	63.0 ± 0.5	62.0 ± 0.5	61.4 ± 0.2	60.9 ± 0.1	
20 - 25	67.4-69.1	65.8-67.9	63.9-65.3	62.8-64.6	57.1-58.8	55.6-56.9	54.9-56.1	54.4-55.0	61.1
	68.1 ± 0.3	66.6 ± 0.3	64.7 ± 0.3	63.6 ± 0.3	58.0 ± 0.5	56.2 ± 0.6	55.5 ± 0.2	54.8 ± 0.1	
25 - 30	64.0-65.7	62.2-64.3	60.2-61.8	58.9-61.1	52.6-54.7	51.4-52.4	50.7-51.6	50.2-50.8	56.6
	64.8 ± 0.4	63.2 ± 0.4	61.1 ± 0.3	59.8 ± 0.4	53.7 ± 0.5	51.9 ± 0.7	51.1 ± 0.2	50.5 ± 0.1	
30 - 40	60.4-61.9	58.6-60.1	56.3-57.5	54.8-57.0	47.4-49.3	45.7-46.9	44.9-45.7	43.9-44.3	50.6
	61.0 ± 0.3	59.2 ± 0.3	56.9 ± 0.3	55.5 ± 0.4	48.5 ± 0.4	46.3 ± 0.5	45.2 ± 0.2	44.1 ± 0.1	
40 - 50	56.5-58.3	54.5-56.2	51.8-53.3	49.9-52.6	41.8-43.6	39.6-40.7	38.5-39.4	37.3-37.7	44.5
	57.2 ± 0.3	55.2 ± 0.3	52.4 ± 0.3	50.8 ± 0.5	42.9 ± 0.3	40.1 ± 0.5	38.9 ± 0.2	37.5 ± 0.1	
50 - 60	53.7-55.4	51.6-53.3	48.7-50.3	46.9-49.4	38.1-39.5	35.7-36.8	34.4-35.3	33.0-33.5	40.2
	54.5 ± 0.4	52.4 ± 0.3	49.5 ± 0.3	47.7 ± 0.5	39.0 ± 0.3	36.2 ± 0.5	34.7 ± 0.2	33.2 ± 0.1	
60 - 70	51.4-53.1	49.2-53.0	46.1-47.7	44.3-46.7	35.1-36.2	32.1-33.3	30.6-31.5	29.2-29.7	36.9
	52.3 ± 0.3	50.1 ± 0.3	46.9 ± 0.3	45.1 ± 0.6	35.8 ± 0.2	32.7 ± 0.4	31.1 ± 0.2	29.4 ± 0.1	
70 - 80	49.5-51.4	47.0-48.9	43.8-45.5	41.6-44.9	32.4-33.6	29.1-30.1	27.4-28.2	25.7-26.3	33.6
	50.3 ± 0.4	47.9 ± 0.4	44.7 ± 0.4	42.7 ± 0.7	33.0 ± 0.3	29.5 ± 0.4	27.8 ± 0.2	26.0 ± 0.1	
80 - 90	48.1-49.9	45.7-47.4	42.2-43.9	40.1-43.5	30.3-31.6	26.9-27.9	25.1-26.0	23.4-23.8	31.2
	49.0 ± 0.4	46.5 ± 0.4	43.1 ± 0.4	41.2 ± 0.7	31.0 ± 0.3	27.4 ± 0.4	25.5 ± 0.2	23.6 ± 0.1	
90 - 100	46.8-48.5	44.2-45.8	40.5-42.2	38.1-41.8	28.1-29.4	24.7-25.4	22.6-23.4	20.7-21.3	28.8
	47.6 ± 0.4	45.0 ± 0.4	41.4 ± 0.4	39.3 ± 0.8	28.8 ± 0.3	25.0 ± 0.5	23.0 ± 0.2	20.9 ± 0.1	
100 - 140	44.6-46.1	42.0-43.5	38.0-39.6	35.3-39.1	24.9-26.1	21.2-22.0	19.2-19.9	17.0-17.3	25.7
	45.3 ± 0.3	42.6 ± 0.3	38.8 ± 0.4	36.6 ± 0.8	25.5 ± 0.2	21.6 ± 0.3	19.5 ± 0.1	17.2 ± 0.1	
140 - 180	42.0-43.9	39.1-41.1	35.2-37.0	32.7-36.9	21.4-22.8	17.5-18.2	15.3-15.9	13.0-13.4	21.4
	43.0 ± 0.4	40.1 ± 0.4	36.2 ± 0.4	33.8 ± 0.9	22.1 ± 0.3	17.8 ± 0.3	15.6 ± 0.1	13.2 ± 0.1	

The length of chromosome 1 is about 247 Mb and there are 39,936 SNPs in this dataset on this chromosome. The numbers of SNP pairs in each distance bin, which would be used to calculate LD, are very large. For every distance bin of 5 kb, there are more than 30,000 SNP pairs used in the calculation for the percent of LD > 0.8, which ensures that the results from our analysis are not due to the effects of small sample sizes.

**Figure 1:**
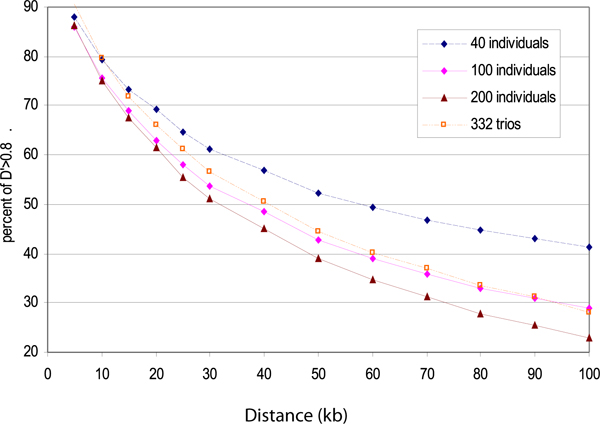
**Mean of estimated percent of D' > 0.8 for different distance bins from 80 samples of independent individuals and estimated percent of D' > 0.8 from the 332 trios**.

Our results show that trios gave quite stable results. In this setting, a sample size of 30 or 40 would give estimates of percent for both *D*' > 0.8 and *r*^2 ^> 0.8 for different distance bins very close to these obtained from 332 trios. However, when independent individuals were used, the estimates were not so stable. When sample size increases, the difference between the estimates from the independent individuals and these from the 332 trios increases. Figure 1 demonstrates this fact. This figure shows the percent of *D*' > 0.8 estimated in data sets with 40, 100, or 200 independent individuals, and 332 trios. As for estimation of percent of *r*^2 ^> 0.8, a similar phenomenon exists, but on a smaller scale.

## Discussion

The two major methods to estimate LD are based on sample of trios and independent individuals. HapMap collected both types of data and used them to estimate LD and established LD maps for different populations. The LD maps help us to plan effective SNP association studies, to effectively locate the disease genes, and to estimate some parameters of population genetics. Also, these data are used to compare the LD pattern across populations.

However, our results show that the estimated LD from independent individuals is not as stable as those from trios. Also, the estimated percent of LD > 0.8 based on independent individuals will be different from those based on the trios. Therefore, we conclude that the estimates from these two designs are not completely comparable under such circumstances. When comparing the LD estimate from these two different sampling methods, we must take this difference into consideration.

There may be some fundamental issues about how to estimate the haplotype and how to use the estimated haplotypes to calculate LD, especially when independent individuals are used. There are two approaches to use the estimated haplotype for individuals. Due to the limited information, we can only estimate the probability for each possible haplotype for each individual. One approach is to assign the haplotype with the highest probability to the particular individual. The other approach is keeping the estimated probability of each possible haplotype, and then use them in the subsequent calculation. These two approaches will give different answers in estimating the LD pattern, especially considering that the first approach will result in loss of information. There is no information on how the haplotypes are used in the calculation of LD in Haploview, so we cannot directly address this issue.

Generally speaking, the trio design, which uses the parents' genotype to infer the offspring haplotype, should give more accurate results. One possible cause of the unstable estimates based on independent individuals is the approach used to estimate haplotype in the LD calculation. Further investigation should be conducted to examine the underlying causes.

## Conclusion

Our results suggest that a trio design is more suitable than using independent individuals in estimating LD. When independent individuals are used, the estimated percents of *D*' > 0.8 and *r*^2 ^> 0.8 are not stable. The estimates using trios design and those using independent individuals are not fully compatible. Caution should be used when comparing LD patterns between a group of independent individuals and a group of trios.

## List of abbreviations used

GAW16: Genetic Analysis Workshop 16; LD: Linkage disequilibrium; SNP: Single-nucleotide polymorphism.

## Competing interests

The authors declare that they have no competing interests.

## Authors' contributions

QH and BJW played an equal role in designing the study and writing the manuscript. QH conducted the analysis.
